# The effect of Setarud (IMOD^TM^) on angiogenesis in transplanted human ovarian tissue to nude mice

**Published:** 2015-10

**Authors:** Maryam Hormozi, Saeed Talebi, Hamid Reza Khorram Khorshid, Amir-Hassan Zarnani, Koorosh Kamali, Mahmood Jeddi-Tehrani, Haleh Soltangoraee, Mohammad Mehdi Akhondi

**Affiliations:** 1 *Biochemistry Department, Lorestan University of Medical Sciences, Khorramabad, Iran.*; 2 *Reproductive Biotechnology Research Center, Avicenna Research Institute, ACECR, Tehran, Iran.*; 3 *Genetic Research Center, University of Social Welfare and Rehabilitation Sciences, Tehran, Iran.*; 4 *Nanobiotechnology Research Center, Avicenna Research Institute, ACECR, Tehran, Iran.*; 5 *Immunology Research Center, Iran University of Medical Sciences, Tehran, Iran.*; 6 *Monoclonal Antibody Research Center, Avicenna Research Institute, ACECR, Tehran, Iran.*

**Keywords:** Angiopoietin, VEGF, Human, Ovary, Setarud

## Abstract

**Background:**

One of the promising methods in fertility preservation among women with cancer is cryopreservation of ovarian cortex but there are many drawbacks such as apoptosis and considerable reduction of follicular density in the transplanted ovary. One solution to reduce ischemic damage is enhancing angiogenesis after transplantation of ovarian cortex tissue.

**Objective:**

The aim of this study was to investigate the effect of Setarud, on angiogenesis in transplanted human ovarian tissue.

**Materials and Methods:**

In this case control study, twenty four nude mice were implanted subcutaneously, with human ovarian tissues, from four women. The mice were randomly divided into two groups (n=12): the experimental group was treated with Setarud, while control group received only vehicle. Each group was divided into three subgroups (n=4) based on the graft recovery days post transplantation (PT). The transplanted fragments were removed on days 2, 7, and 30 PT and the expression of Angiopoietin-1, Angiopoietin-2, and Vascular endothelial growth factor at both gene and protein levels and vascular density were studied in the grafted ovarian tissues.

**Results:**

On the 2^nd^ and 7^th^ day PT, the level of Angiopoietin-1 gene expression in case group was significantly lower than that in control group, while the opposite results were obtained for Angiopoietin-2 and Vascular endothelial growth factor. These results were also confirmed at the protein level. The density of vessels in Setarud group elevated significantly on day 7 PT compared to pre-treatment state.

**Conclusion:**

Our results showed that administration of Setarud may stimulates angiogenesis in transplanted human ovarian tissues, although further researches are needed before a clear judgment is made.

## Introduction

Many cancer patients become infertile after treatment and this can be an important concern for younger people. One of the promising methods to restore fertility of women patients is cryopreservation of ovarian cortex before staring cancer treatment. There are many drawbacks such as apoptosis and considerable reduction of follicular density in the transplanted ovary (1-3). Some studies have shown that these problems mainly result from ischemic damages and not from freezing/thawing process of the tissue (1). One solution to reduce ischemic damage is enhancing angiogenesis after transplantation of ovarian cortex tissue (1, 4).

It seems that various angiogenic factors are expressed in the ovary. Vascular endothelial growth factor (VEGF) and angiopoietins (Ang) due to their specific effects on endothelial cells have more important roles (5-10).

VEGF has a crucial role in angiogenesis by acting through migration, proliferation, and differentiation of endothelial cells, formation of immature veins, and vascular permeability (1, 5, 11). Angiopoietin, another family of growth factors, has an important role in effective function of VEGF, remodeling, branching, maturation, and perseverance of veins through interaction with extra cellular matrix (5, 7-10, 12, 13). 

Setarud (IMOD^TM^) is a mixture of herbal extract of different plants (Tanacetum vulgare, Rosa canina, Urtica dioica) which various studies have shown its beneficial effects on immune system, lipid metabolism, liver function, and inflammatory processes (14-18). Setarud contains various compounds such as selenium, beta carotene and tannin that some studies have shown their effects on angiogenesis (19-26).

Selenium as selenomethionine has potent angiogenic effects in the corneal pocket or chorioallantoic membrane assays. It was shown that selenium induces migration and proliferation of aortic cells leading to a three-fold increase in cell proliferation (24).

There are reports on the effect of beta-carotene on the stimulation of angiogenesis. It can induce angiogenic gene expression and promotes cell differentiation by binding to nuclear retinoid receptors (20, 21, 25, 27). Tannin has also angiogenic activity. It seems that tannin increases the amount of newly formed capillaries by up-regulating VEGF expression (22).

Since successful transplantation of ovarian cortex is dependent on the reduction of apoptosis and maintenance of follicular density and angiogenesis is one of the best solutions for inhibiting ischemic damages, the aim of this study was to assess the effect of Setarud on the promotion of angiogenesis in transplanted fragments of human ovary to nude mice by measuring the level of gene and protein expression of VEGF, Ang-1 and Ang-2 which are effective in angiogenesis and evaluation of vascular density.

## Materials and methods

**Experimental design**

This case control study was conducted at Avicenna Research Institute in order to assess the potential effects of Setarud (IMOD^TM^) on induction of angiogenesis in transplanted human ovarian tissues. Human ovarian fragments were grafted subcutaneously to twenty four mature (aged 6 to 8 weeks) female B6cg nude mice. The mice were then randomly divided into two groups (n=12 mice per group) of case and control: the case group was treated with Setarud 1 ml/kg (30 mg/ml), (Rose Pharmed Co., Iran), every day, while control group received only vehicle (8.6 % ethanol v/v) (Merck, Darmstadt, Germany). The animals in each group were divided into three subgroups of 4 mice each based on the graft recovery days post transplantation (PT). All injections were performed as intravenous (IV) from day 1 until day 7 for both groups and after that until day 30 were injected subcutaneously. The grafts were recovered from the subgroups on the 2nd, 7th or 30th day after transplantation.

**Ovarian samples**

The use of human tissue for this study was approved by the local ethics committee of Avicenna Research Institute. Ovarian biopsy specimens were taken from four women (between 23 and 38 years of age), after obtaining written informed consent. They were all undergoing surgery for sex reversal or ovarian cyst. They did not have endometriosis or other ovarian diseases.

In the operating room, immediately after biopsy specimen retrieval, ovarian tissue was immersed in a solution of Dulbecco´s minimum essential medium (Sigma, St. Louis, Missouri, USA) supplemented with 10% human serum and transported to the laboratory on ice. Each ovarian biopsy specimen was cut into pieces of 2-3 mm^3^ and implanted to nude mice SC.

**Animals**

Twenty-four 8 to 10 weeks-old female B6cg nude mice weighing between 20-30 g were obtained from Tehran University of Medical Sciences. The animals were housed under specific pathogen-free conditions with a constant temperature (22-25^º^C), relative humidity (55%) and 12-h dark/light cycles. All procedures, tests, and injections were performed under a laminar flow hood in a positive pressure room. Approval for the study was obtained from the local ethical committee on animal experiments. The animals were maintained in accordance with Animal Care and Use Committee Regulations.

**Anesthesia and ovarian transplantation**

The mice were anesthetized by intraperitoneal injection of ketamine (Alfasan, Woerden, NL) 100 mg/Kg and Xylazine (Alfasan) 10 mg/Kg. The incision site was thoroughly disinfected with 70% ethanol (Merck) and betadine. Human ovarian pieces were grafted under the back skin of each mouse (28, 29). The mice were sacrificed 2, 7, and 30 days PT and ovarian fragments were removed from each animal. One fragments of transplanted ovarian tissue was immersed in 10% formalin (Merck) for histological evaluation and the others were frozen in liquid nitrogen and then stored at -70^º^C for molecular evaluations.

**Histological evaluation**

Briefly, fixed ovarian fragments in 10% formalin were processed into paraffin blocks and then specimens were cut into serial sections with 3-5 micron thickness. Moreover, they were stained according to Hematoxylin and Eosin (Merck) (H & E) staining protocol. The number of single layer vessels for each tissue was counted in at least 5 sections in high power field (HPF) and averaged and averaged. The data for each experimental group was shown as the mean density of vessels per microscopic field at 400×magnification (vessel number/HPF).

**Analysis of VEGF, Ang-1, and Ang-2 gene expression by real time PCR**

Total RNA was extracted using a Trizol reagent (Invitrogen Life Technologies, Carlsbad, CA, USA) according to the manufacturer’s instruction. The final RNA pellet was washed with 75% ethanol, air dried, and then dissolved in diethy l pyrocarbonate treated water (CinnaGen, Tehran, Iran). The concentration and purity of RNA was determined by biophotometer (Eppendorf, Hamburg, Germany). First strand cDNA was generated with 1 µg total RNA using the cDNA Synthesis Kit (Roche Diagnostics GmbH, Mannheim, Germany). The house keeping gene for normalization was Hypoxanthine guanine phosphoribosyl transferase (HPRT). Real-time quantitative PCR was performed using ABI 7500 real time PCR system and SYBR-Green Premix Ex Taq kit (TAKARA, Otsu, Shiga, Japan). The reactions were performed with the following settings: 95^°^C for 10 s (initial denaturation), 40 cycles of denaturation at 95^°^C for 5 s, and annealing at 56^°^C for 30 s (for Ang-1 and Ang-2), or 60^°^C for 30 s (for HPRT and VEGF), and extension at 72^°^C for 30 s each. 

**Western blot analysis**

The extraction of protein from ovarian fragments was down on ice with lysis buffer containing (Tris-HCl 20 mM, NaCl 137 mM, glycerol 10% v/v, NP40 1% v/v, EDTA 2 mM, and protease inhibitor cocktail (Sigma). Protein lysates were then centrifuged and their protein concentrations were determined using BCA protein assay kit (Thermo Scientific Pierce, Rockford, IL, USA). The extracted proteins were resolved on a 12% SDS-PAGE and transferred to PVDF membranes. After blocking by incubating the membranes with blocking solution containing 5% non-fat milk overnight at 4^°^C, the membranes were incubated with human-specific primary antibodies including rabbit anti-VEGF (Abcam, Cambridge, UK), goat anti-Ang-1 (Sigma), rabbit anti-Ang-2 (Abcam) or rabbit anti-β-actin (Abcam) diluted 1: 1000 in 3% non-fat milk for 2 hr at room temperature. The membranes were washed in Tris-buffered saline with 0.05% Tween-20 and then incubated with peroxidase-conjugated sheep anti-rabbit Ig (Avicenna Research Institute, Tehran, Iran) diluted 1: 3500, or rabbit anti-goat Ig (Razi BioTech, Tehran, Iran) diluted 1:4000 in 3% non-fat milk for 1 hr at room temperature. The peroxidase activity was visualized using an enhanced chemiluminescence (ECL) kit (GE Health care, Uppsala Sweden) and band densities were analyzed using AlphaEase software. The relative expression levels of proteins (VEGF, Ang-1 and Ang-2) were indicated by comparing density of each band with that of the internal control, β-actin.

**Statistical analysis**

The total expression ratio of the genes of interest at three time points was compared between Setarud (IMOD^TM^) and control groups using a randomization test implemented in the relative expression software tool (REST), which is an Excel-based application for comparison and statistical analysis of relative expression results in qRT-PCR (30). The same software was also used for group wise comparison of the gene expression between each group and pre-treatment state. Vascular densities in the two groups were compared by Wilcoxon signed rank test using SPSS11.5 software (Statistical Package for the Social Sciences, SPSS Inc, Chicago, Illinois, USA). Differences were considered significant when p<0.05.

## Results

**Analysis of vascular density**

**Figure 1.**
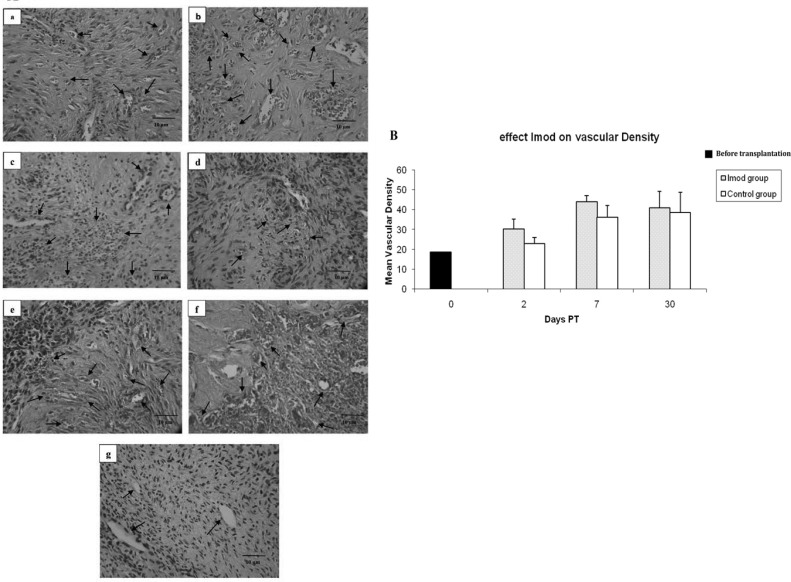
A) Representative photographs of transplanted human ovarian tissue showing vascular density in Setarud (IMOD^TM^) and control groups. Nude mice were treated with Setarud (a-c) or vehicle (d-f) after being transplanted with human ovarian tissues. Tissues were recovered on days 2 (a, d), 7 (b, e), or 30 (c, f) PT (post transplantation) and evaluated for vascular density after H & E staining. g: pre-transplantation ovarian tissue. Black arrows show blood vessels.(B) Evaluation of vascular density in transplanted human ovarian tissue in Setarud (IMOD^TM^) and control groups on days 2, 7 and 30 post transplantation compared to before transplantation (black column).

Evaluation of vascular density using nonparametric test showed that the density of vessels in Setarud (IMOD^TM^) group elevated significantly at day 7 PT compared to pre-treatment state (p=0.043) (Figures 1-A and B).

**Ang-1 expression**

**Figure 2:**
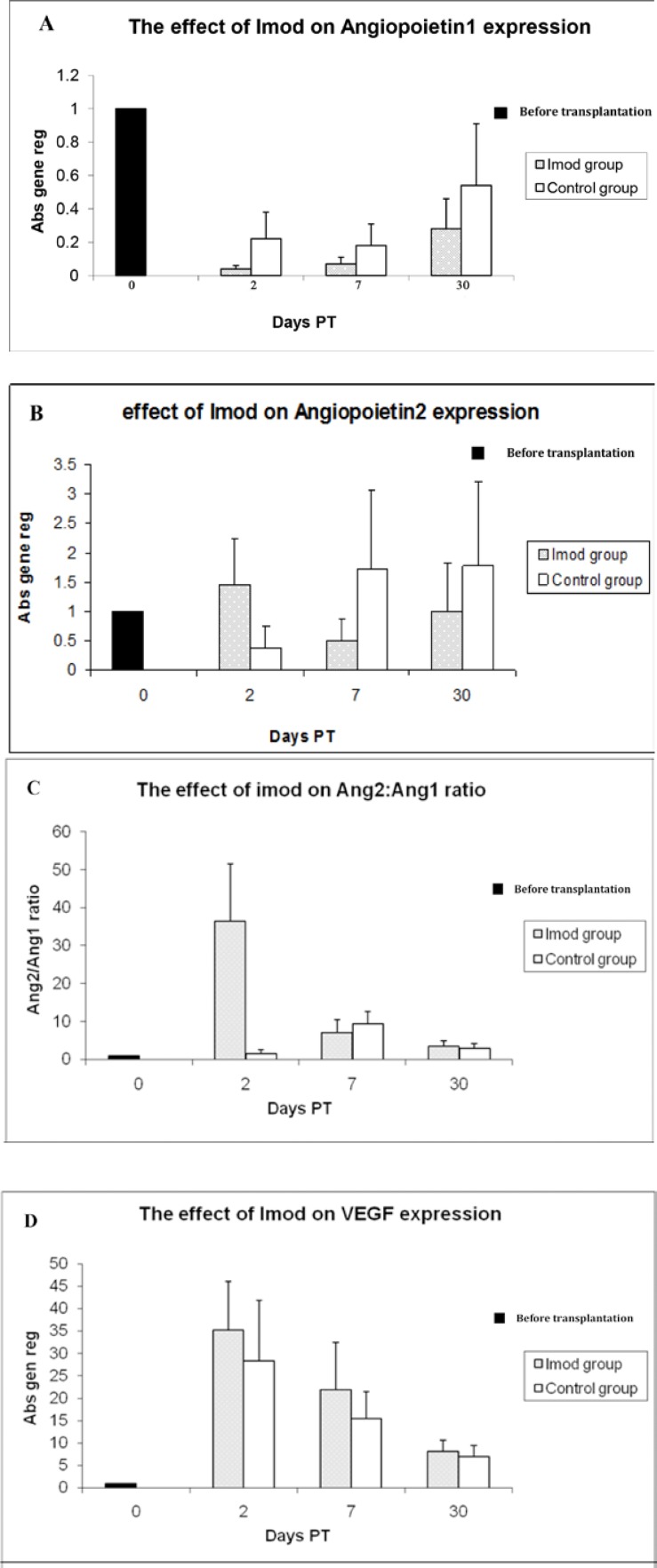
Relative expression of Angiopoietin-1, Angiopoietin-2, and Vascular endothelial growth factor genes in transplanted human ovarian tissues in Setarud (IMOD^TM^) and control groups at different time intervals after transplantation. Nude mice were treated with Setarud or vehicle after transplantation with human ovarian tissues. On days 2, 7 and 30 post transplantation, ovarian fragments were removed and the expression of Ang-1 (A), Ang-2 (B), Ang-1/Ang-2 ratio (C) and VEGF (D) were assessed by quantitative real time PCR. All comparisons were made compared to pre-treatment state (0). ٭p<0.05, ٭٭p<0.01 , Abs gene reg: Absolute gene regulation

**Figure 3:**
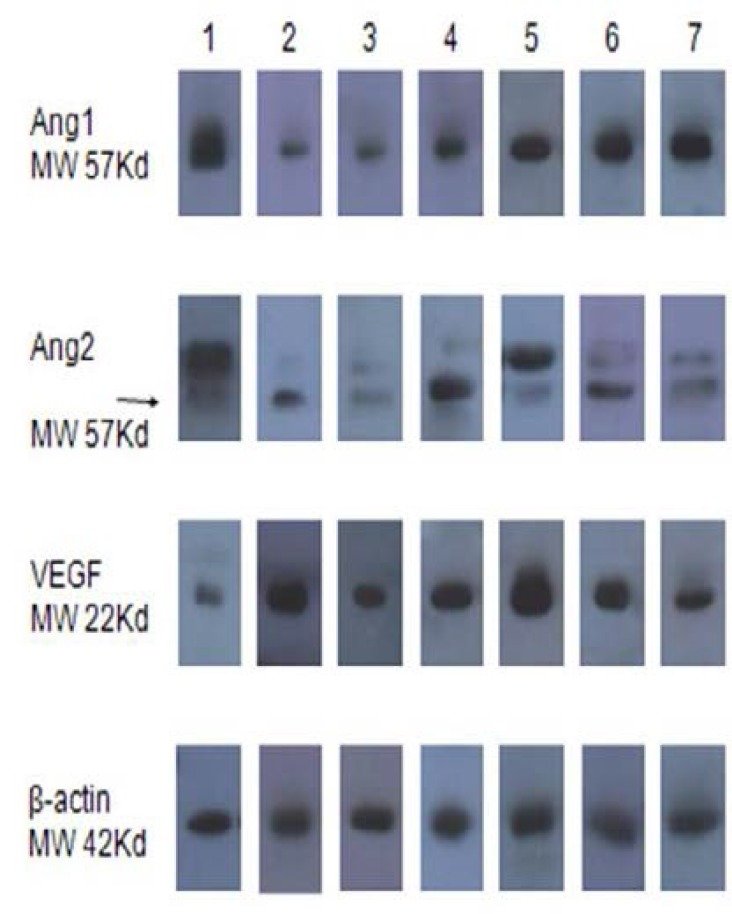
Western blot analysis of ovarian Angiopoietin-1, Angiopoietin-2, and Vascular endothelial growth factor expression in Setarud (IMOD^TM^), and control groups.

Analysis of Ang-1 gene expression by real-time RT-PCR showed significant decrease in the Setarud (IMOD^TM^) group on days 2, 7 and 30 compared to pre-treatment state (p=0.001, p=0.001 and p=0.038 respectively). In control group, the gene expression rate revealed to be significantly lower only on day 7 PT as compared to pre-treatment state (p=0.001) (Figure 2-A). Compared to the control group, the level of Ang-1 gene expression on days 2 and 7 were significantly lower in Setarud group (p=0.007 and p=0.008 respectively), (Table I). Data obtained by Western blot analysis showed the similar pattern of Ang-1 protein expression in both groups and time intervals were examined (Figure 3).

**Ang-2 expression**

Gene expression of Ang-2 in Setarud (IMOD^TM^) and control groups showed opposite patterns throughout the study (Figure 2-B); the statistical analysis, however, did not show any significant variation of the gene in both groups compared to pre-treatment state. Comparison between the Setarud (IMOD^TM^) and control groups showed significant increase in gene expression on days 2 PT (p=0.007) and lower gene expression on day 7 PT (p=0.028) in Setarud group (Table I). The pattern of Ang-2 protein expression followed that of Ang-2 gene, but no strict consistency at all time periods was observed (Figure 3).

**Ang-2: Ang-1 expression**

It seems that evaluation of Ang-1 or Ang-2 separately did not give a precise picture of their contribution in angiogenesis but their ratio might be more important. With this assumption, the Ang-2: Ang-1 ratio was calculated for each PT time interval and was compared between the control and Setarud groups. It was surprising that this ratio was found to be significantly higher (p=0.001) in the Setarud (IMOD^TM^) group compared with the control group on day 2 PT. This ratio was inverted on day 7 PT (p=0.03) (Figure 2-C). 

**VEGF expression**

Expression rate of VEGF gene in Setarud (IMOD^TM^) group elevated significantly on days 2, 7 and 30 PT in comparison to pre-treatment state (p=0.001, p=0.001 and p=0.021, respectively), (Figure 2-D). The VEGF was significantly up-regulated on days 2 and 7 in the Setarud (IMOD^TM^) group compared with the control group (p=0.045 and p=0.045, respectively), (Table I). The pattern of VEGF protein expression (Figure 3) was in line with what was found at the gene level (Figure 2-D).

**Table I:** The total expression ratio of the genes of interest in Setarud (IMOD^TM^) group relative to control group is presented at each time point (2^nd^, 7^th^ and 30^th^ day after transplantation)

	**Day 2 PT**			**Day 7 PT**			**Day 30 PT**		
	**Ang-1**	**Ang-2**	**VEGF**	**Ang-1**	**Ang-2**	**VEGF**	**Ang-1**	**Ang-2**	**VEGF**
Relative expression	0.15	3.74	1.24	0.33	0.30	1.42	0.450	0.45	1.16
SEM	0.11	3.8	0.89	0.24	0.28	0.51	0.34	0.41	0.65
p-value	0.007	0.007	0.045	0.008	0.028	0.045	0.233	0.254	0.065
Fold increase/decrease	-6.64	3.74	1.24	-3.02	-3.31	1.42	-2.22	-2.23	1.16

Significant down- or up-regulations of the genes are highlighted.

PT: Post transplantation; SEM: Standard error of mean; Ang-1: Angiopoietin-1; Ang-2: Angiopoetin-2; VEGF: Vascular endothelial growth factor

* Pair-Wise fixed Reallocation Randomization Test (Permutation test with 2000 iterations)

Lane1: pre-treatment ovary

Lanes 2-4: days 2, 7, 30 post transplantation in Setarud (IMOD^TM^) group, respectively

Lanes 5-7: days 2, 7, 30 post transplantation in control group, respectively

## Discussion

Our results showed a beneficial effect of Setarud (IMOD^TM^) on triggering angiogenesis process in heterptopic transplanted ovarian cortex leading to inhibition of ischemic damages and preservation of follicular density.

Data on gene expression level of Ang-1 clearly showed gene down regulation in Setarud (IMOD^TM^) group on 2nd and 7th days in which these down-regulations were significantly lower than vehicle-treated ovaries. Studies have shown that Ang-1 has anti-inflammatory and anti-apoptotic effects on endothelial cells during the plastic phase of angiogenesis (31-36). Reportedly, in plastic phase of angiogenesis, the decrease in the level of Ang-1 gene expression resulted in loss of integrity of existing vasculature. This process may trigger the development of newly formed vessels (7). It seems that Setarud (IMOD^TM^) stimulates angiogenesis and induction of new blood vessels maturation by significant reduction in Ang-1 expression soon after transplantation. In contrast to the expression level of Ang-1, the expression of Ang-2 on days 2 and 7 were higher in Setarud (IMOD^TM^) group compared to the control group. It has been shown that increase of Ang-2 to Ang-1 ratio causes loss of vascular integrity and consequently stimulation of angiogenesis (7, 8, 37).

The pattern of VEGF gene expression during the study period was similar in both groups with initial increase followed by decrease and there was a significant difference between the two groups, which suggests apparent effect by Setarud on VEGF expression. There are many reports on the synergistic effect of VEGF and Ang-2 on the stimulation of angiogenesis (5, 6, 8-10, 12, 38-51), suggesting a beneficial effect of Setarud (IMOD^TM^) on initiation of angiogenesis.

The results of Western blot analysis of protein, Ang-1, Ang-2 and VEGF confirmed the pattern of their gene expression, although, in some time intervals no strict consistency was observed which may be related to small number of studied cases. Setarud (IMOD^TM^) treatment also significantly increased the vascular density at day 7 PT with the normalization of neovascularization on day 30 PT. Therefore, it seems that simultaneous increment of Ang-2 and VEGF expression in the Setarud group may accelerate angiogenesis process shortly after transplantation. However, further studies with more samples are needed for confirmation of this hypothesis.

Setarud (IMOD^TM^), as the extract of three plants, contains several pharmaceutical active compounds. The anti-oxidant effects of this drug have been documented by other studies (14, 15). Such consequence may also be responsible for the beneficial effects observed in our study. It has been shown that selenium, beta-carotene and tannin as the active components of the extract can trigger angiogenesis (19-26). Since no report on the differential effects of each component of the extract is available, it cannot be determined confidently which components or their combination is responsible for the stimulation of angiogenesis.

## Conclusion

In conclusion, our data suggest that Setarud (IMOD^TM^) induces angiogenesis and reduces ischemic damages in heterotopic transplanted human ovarian tissue.
